# Increased Mutability of Staphylococci in Biofilms as a Consequence of Oxidative Stress

**DOI:** 10.1371/journal.pone.0047695

**Published:** 2012-10-24

**Authors:** Victoria J. Ryder, Ian Chopra, Alex J. O’Neill

**Affiliations:** Antimicrobial Research Centre and School of Molecular and Cellular Biology, Faculty of Biological Sciences, University of Leeds, Leeds, United Kingdom; National Institutes of Health, United States of America

## Abstract

**Objectives:**

To investigate the development of mutational resistance to antibiotics in staphylococcal biofilms.

**Methods:**

Mutation frequencies to resistance against mupirocin and rifampicin were determined for planktonic cultures and for biofilms generated using either a novel static biofilm model or by continuous flow. DNA microarray analysis was performed to detect differences in transcriptional profiles between planktonic and biofilm cultures.

**Results:**

The mutability of biofilm cultures increased up to 60-fold and 4-fold for *S. aureus* and *S. epidermidis,* respectively, compared with planktonic cultures. Incorporation of antioxidants into *S. aureus* biofilms reduced mutation frequencies, indicating that increased oxidative stress underlies the heightened mutability. Transcriptional profiling of early biofilm cultures revealed up-regulation of the superoxide dismutase gene, *sodA*, also suggestive of enhanced oxidative stress in these cultures. The addition of catalase to biofilms of *S. aureus* SH1000 reduced mutation frequencies, a finding which implicated hydrogen peroxide in increased biofilm mutability. However, catalase had no effect on biofilm mutability in *S. aureus* UAMS-1, suggesting that there is more than one mechanism by which the mutability of staphylococci may increase during the biofilm mode of growth.

**Conclusion:**

Our findings suggest that biofilms represent an enriched source of mutational resistance to antibiotics in the staphylococci.

## Introduction

In the natural environment bacteria frequently associate with surfaces [1]. This adherent mode of growth, usually associated with extracellular matrix production, is known as a biofilm. The biofilm growth state is also adopted by members of the human microbiome and by bacterial pathogens during a variety of acute and chronic infections [2], [3]. Biofilm-associated infections are notoriously difficult to eradicate due to the refractory nature of organisms in this growth state which are able to resist killing by the majority of antibiotics and the immune system [4], [5], [6], [7], [8]. Furthermore, there is limited but growing evidence to suggest that biofilms might facilitate the emergence of antibiotic resistance. Enhanced rates of conjugation have been reported in biofilms of enterococci and *Pseudomonas* spp [9], [10] and increased mutation frequencies to antibiotic resistance have been detected in biofilms of *Pseudomonas aeruginosa* and *Streptococcus pneumoniae* [11], [12].

Staphylococci cause an array of human infections which involve a biofilm component, including osteomyelitis, endocarditis, and infections associated with indwelling medical devices [13], [14], [15], [16]. It is unknown whether the staphylococcal biofilm might facilitate the emergence of resistance to antibacterial agents. Therefore we examined whether staphylococci become more mutable during the biofilm mode of growth compared with planktonic culture. The majority of established biofilm models are low throughput in nature, as with most flow systems [11], [17], whilst those that are high throughput (e.g. those generated using microtitre plate models) yield biofilms with comparatively low cell densities [18]. Therefore for this study we developed and validated a new static biofilm model to overcome the limitations of the existing approaches. The biofilms generated using our method achieved a yield of cells sufficient for mutation frequency determinations, and enabled biofilm culture for prolonged periods to permit investigation of the mutability of biofilms of differing maturity.

## Results

### Mutability of Staphylococcal Biofilms

**Figure 1:**
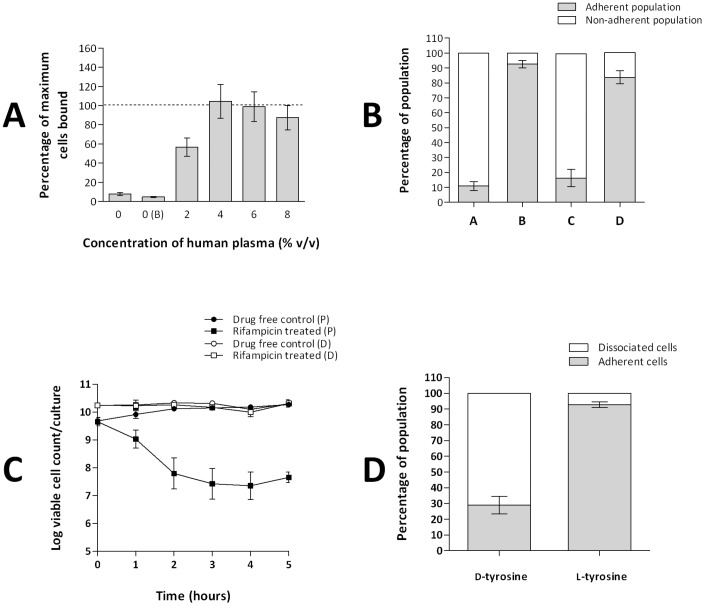
Development and validation of the cellulose disk static biofilm model. **A**. Effect of human plasma on the adherence of *S. aureus* SH1000 to cellulose disks. On the x-axis, 0 represents no human plasma or buffer and 0(B) is buffer only. Data indicate the proportion of adherent cells, in 48 hr disk cultures, as a percentage of the maximum achieved at 10% (v/v) human plasma (dashed line) (1.8×10^10^ cfu/disk). Data from three experimental replicates. Error bars indicate standard error. **B.** Determination of the proportion of adherent and planktonic cells present in *S. aureus* SH1000 cellulose disk cultures. (A) Cultures grown for 48 hrs in the absence of human plasma. (B) Cultures grown for 48 hrs with human plasma (4% v/v) added prior to inoculation. (C) Cultures grown for 144 hrs with human plasma (4% v/v) added prior to inoculation. (D) Cultures grown for 144 hrs with human plasma (4% v/v) added prior to inoculation and every 48 hrs. Data from three experimental replicates. Error bars indicate standard error. **C.** Time-kill curves of *S. aureus* SH1000 exponential phase planktonic (P) and 48 hr cellulose disk (D) cultures exposed to 0.25 mg/L rifampicin. Data from three experimental replicates. Error bars indicate standard deviations. **D.** The dissociation of adherent cells of *S. aureus* SH1000 from cellulose disk cultures in the presence of d-tyrosine (100 µM) and l-tyrosine (100 µM). Data from three experimental replicates. Error bars indicate standard error. Methodology and additional information regarding validation of the cellulose disk model can be found in Supporting Information S1.

The limitations of existing biofilm models for performing mutation frequency determinations led us to develop a novel cellulose disk static (CDS) biofilm model better suited for this purpose. Although static biofilm models utilising membrane disks have previously been described [19], [20], they have to our knowledge not been widely used for culture of staphylococcal biofilms, nor have they been validated for this purpose. Extensive validation of the CDS model was performed to ensure that biofilms were indeed formed, including demonstration of cell adherence which was reversed in the presence of d-amino acids [21], [22] (Figure 1A, 1B and 1D), confirmation that bacteria associated with the filters exhibited recalcitrance to antibiotic-mediated killing (Figure 1C) and visualisation of biofilms by confocal and atomic force microscopy (*data not shown*). A description of the methods and results of the validation experiments can be found in Information S1.

**Figure 2:**
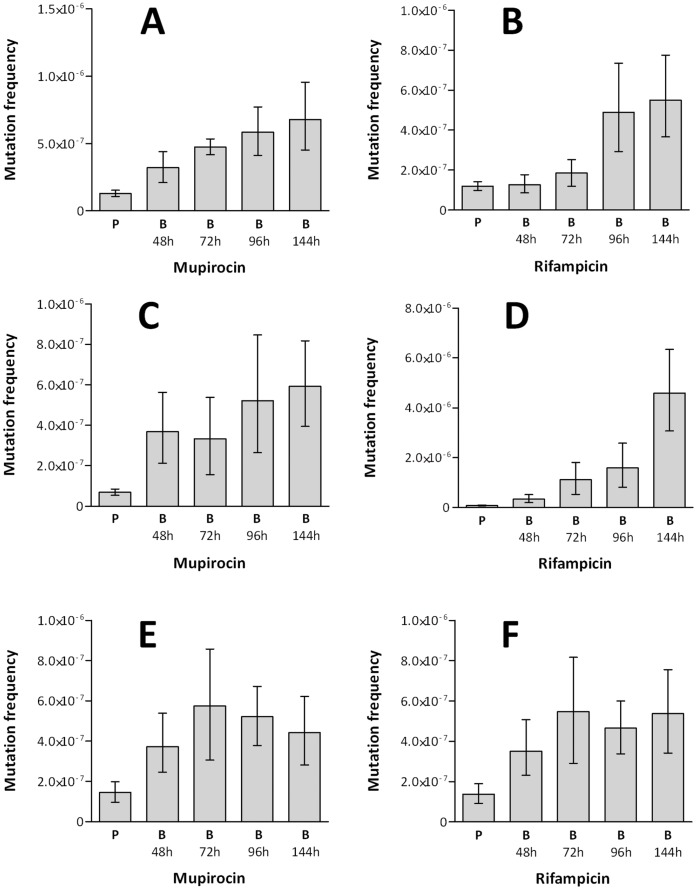
Mutation frequencies of static staphylococcal biofilms. Mutation frequencies of planktonic and biofilm cultures (grown using the cellulose disk biofilm model) of *S. aureus* SH1000 (**A** & **B**), *S. aureus* UAMS-1 (**C** & **D**) and *S. epidermidis* RP62A (**E** & **F**), determined by selection of spontaneous mutants resistant to mupirocin (**A**, **C** & **E**) or rifampicin (**B**, **D** & **F**). P indicates planktonic cultures and 48 h, 72 h, 96 h and 144 h indicate biofilm (B) incubation time. Data from six experimental replicates. Error bars indicate 95% confidence intervals.

Mutation frequencies were determined for *S. aureus* SH1000 grown in planktonic cultures in MHB for 18 hrs, and for CDS biofilms of various maturation states, from 48 to 144 hrs. Compared with planktonic cultures, maximum mutation frequency increases of 4.1-fold and 4.6-fold were observed in the 96 hr biofilm cultures, using rifampicin and mupirocin selection, respectively (Figure 2A and 2B). To confirm that this increase in mutation frequency was not the result of using a different growth media in the biofilm cultures (BHA) compared with planktonic cultures (MHB), or as a consequence of the extended periods of incubation to which the biofilms were subjected, we also determined the mutation frequency of planktonic cultures of *S. aureus* SH1000 incubated for 96 hrs in BHB; no significant difference in mutability was observed for these cultures compared with planktonic cultures grown for 18 hrs in MHB (*data not shown*).

**Figure 3:**
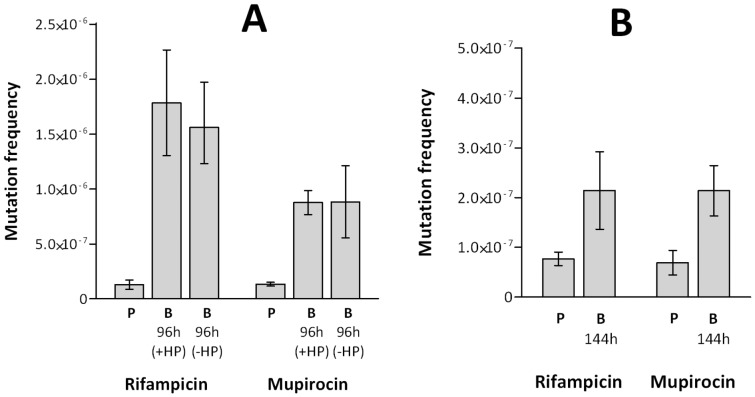
Mutation frequencies of *S. aureus* biofilms grown under constant flow. Mutation frequencies of planktonic and biofilm cultures (grown in the Sorbarod model) of *S. aureus* SH1000 (**A**) and *S. aureus* UAMS-1 (**B**) determined by rifampicin and mupirocin selection. P indicates planktonic cultures and 96 h and 144 h indicate biofilm (B) incubation time. Biofilm cultures were incubated in the presence (+HP) and absence (−HP) of 4% (v/v) human plasma. Data are based on three experimental replicates. Error bars indicate 95% confidence intervals.

To examine whether increased mutability is associated with the biofilm mode of growth in other strains of *S. aureus*, equivalent experiments were performed with the prolific biofilm-forming strain, UAMS-1 [23]. CDS biofilms of UAMS-1 exhibited an even greater increase in mutability over planktonic cultures compared to SH1000; maximum mutability was observed in biofilms grown for 144 hr, at which point increases in mutation frequency to rifampicin and mupirocin resistance increased 59.5-fold and 8.6-fold, respectively (Figure 2C and 2D). Increased mutability in biofilms could also be demonstrated in staphylococci other than *S. aureus.* Mutation frequency determinations for planktonic and CDS biofilm cultures of *S. epidermidis* RP62A revealed maximal mutability after 72 hrs of biofilm growth, at which point mutation frequencies to rifampicin and mupirocin resistance were 4.0-fold greater than observed with planktonic cultures (Figure 2E and 2F).

**Figure 4:**
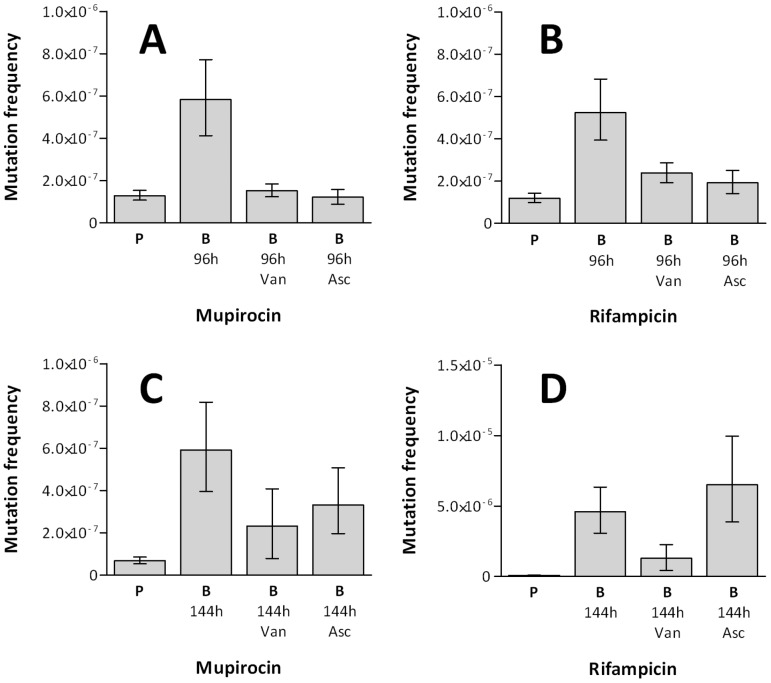
The effect of antioxidants on *S. aureus* biofilm mutability. Mutation frequencies of *S. aureus* SH1000 (**A** & **B**) and UAMS-1 (**C** & **D**) planktonic (P) and biofilm (B) cultures in the presence of antioxidants vanillin (Van) and ascorbic acid (Asc). Mutation frequencies were determined using mupirocin (**A** & **C**) and rifampicin (**B** & **D**) selection. Data from six experimental replicates. Error bars indicate 95% confidence intervals.

To ensure that the observed increases in mutability were neither unique to static biofilms formed in the absence of shear forces, nor an artefact of the CDS biofilm model, mutation frequencies to antibiotic resistance were also determined for biofilms of *S. aureus* SH1000 and UAMS-1 cultured using the Sorbarod flow system. Furthermore, to exclude the possibility of an impact on mutability by the human plasma used to prime the substratum for maximum biofilm yield in the CDS biofilm model, these experiments were conducted both in the presence and absence of human plasma. Sorbarod biofilms were grown for 96 hrs (SH1000) and 144 hrs (UAMS-1), since the greatest increases in mutability were observed at these time-points in the CDS model. *S. aureus* SH1000 Sorbarod biofilms exhibited 14.4-fold and 6.4-fold increases in mutation frequency to rifampicin and mupirocin resistance, respectively, compared with planktonic cultures (Figure 3A). Mutation frequency increases of 2.2-fold (rifampicin) and a 3.1-fold (mupirocin), compared with planktonic cultures, were also observed in *S. aureus* UAMS-1 Sorbarod biofilms (Figure 3B). Biofilms grown in the presence or absence of plasma exhibited no significant differences in mutation frequency (Figure 3A).

**Table 1:** Differentially regulated genes in *S. aureus* SH1000 biofilms, grown for 48 hrs and 144 hrs, compared with planktonic cultures. ORFs encoding hypothetical proteins not shown.

ORF	Gene	Function	Fold change
**48 hr Biofilms/Planktonic**			
SAOUHSC_00502	*ctsR*	Transcriptional regulator CtsR	2.20 up
SAOUHSC_00526		Putative ribosomal protein L7Ae-like	2.30 up
SAOUHSC_00894	*rocD2*	Ornithine aminotransferase 2	2.61 up
SAOUHSC_00895		Glutamate dehydrogenase, NAD-specific, putative	2.36 up
SAOUHSC_01002	*qoxA*	Probable quinol oxidase subunit 2	2.00 up
SAOUHSC_01042		Dihydrolipoamide S-acetyltransferase component of pyruvate dehydrogenase complex E2, putative	2.17 up
SAOUHSC_01103		Succinate dehydrogenase cytochrome b-558 subunit, putative	2.16 up
SAOUHSC_01216	*sucC*	Succinyl-CoA synthetase subunit beta	2.44 up
SAOUHSC_01389	*pstS*	Phosphate-binding protein PstS	2.24 up
SAOUHSC_01407		Putative XpaC protein	2.04 up
SAOUHSC_01408		TelA-like protein	2.32 up
SAOUHSC_01418	*odhA*	2-oxoglutarate dehydrogenase E1 component	2.02 up
SAOUHSC_01462	*gpsB*	Cell cycle protein gpsB	2.37 up
SAOUHSC_01477		Putative Zn dependent protease	2.38 up
SAOUHSC_01653	*sodA*	Superoxide dismutase [Mn] 1	2.77 up
SAOUHSC_01683	*dnaK*	Chaperone protein DnaK	2.47 up
SAOUHSC_01684	*grpE*	Protein GrpE	3.15 up
SAOUHSC_01685	*hrcA*	Heat-inducible transcription repressor HrcA	2.84 up
SAOUHSC_01720		Putative Holliday junction resolvase	2.00 up
SAOUHSC_01801	*icd*	Isocitrate dehydrogenase	2.26 up
SAOUHSC_01880		Transposase domain protein	3.32 down
SAOUHSC_02261	*agrB*	Accessory gene regulator protein B	2.76 up
SAOUHSC_02262	*agrD*	Accessory gene regulator protein D	3.56 up
SAOUHSC_02264	*agrC*	Accessory gene regulator protein C	2.62 up
SAOUHSC_02350	*atpB*	ATP synthase subunit A	2.03 up
SAOUHSC_02488	*rpmJ*	50S ribosomal protein L36	2.23 up
**144 hr Biofilms/Planktonic**			
SAOUHSC_01683	*dnaK*	Chaperone protein DnaK	2.17 up
SAOUHSC_01684	*grpE*	Protein GrpE	2.47 up
SAOUHSC_01685	*hrcA*	Heat-inducible transcription repressor HrcA	2.43 up
SAOUHSC_02260	*hld*	Delta hemolysin	3.55 down
SAOUHSC_02466		Truncated MHC class II analog protein	2.34 down

### Oxidative Stress Contributes to Increased Biofilm Mutability

Previous studies have suggested that oxidative stress in *P. aeruginosa* and *S. pneumoniae* biofilms might prompt phenotypic and genotypic variation [11], [12], [24]. To investigate whether oxidative stress has a role in increasing mutability of staphylococcal biofilms, we examined the impact on mutation frequencies to antibiotic resistance of incorporating the antioxidants vanillin and ascorbic acid into biofilm cultures at ¼×MIC. CDS biofilms of *S. aureus* SH1000 demonstrated significant reductions in mutation frequency in the presence of either antioxidant compound (Figure 4A and 4B); in some cases the frequency of mutation decreased to that seen with planktonic cultures. Similarly, biofilms of *S. aureus* UAMS-1 demonstrated reduced mutation frequencies to mupirocin resistance for both antioxidants tested; however, only vanillin reduced the mutation frequency to rifampicin resistance (Figure 4C and 4D). By contrast, the addition of antioxidants to planktonic cultures of SH1000 or UAMS-1 did not reduce the basal mutation frequency to antibiotic resistance (*data not shown*).

**Figure 5:**
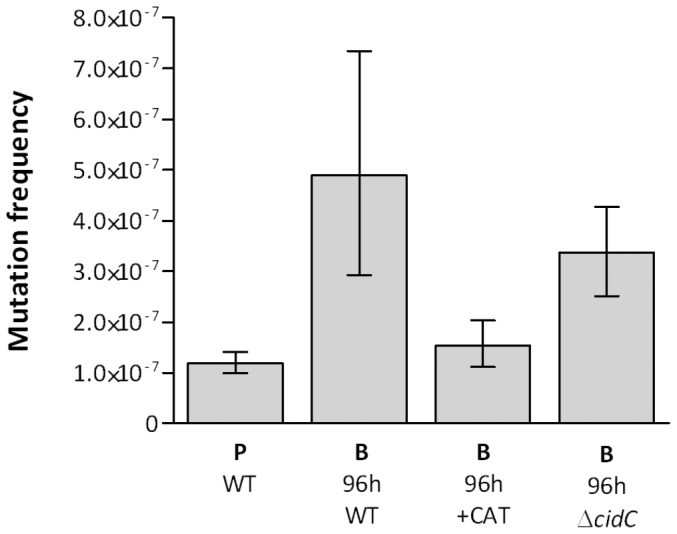
The effect of catalase and *cidC* inactivation on *S. aureus* biofilm mutability. Mutation frequencies of *S. aureus* SH1000 planktonic (P) and 96 h biofilm (B) cultures, determined by rifampicin selection. WT indicates wild-type (*S. aureus* SH1000), +CAT indicates wild-type supplemented with catalase (4 U/ml) and *ΔcidC* indicates a CidC-deficient mutant of *S. aureus* SH1000. Data from six experimental replicates. Error bars indicate 95% confidence intervals.

In *P. aeruginosa* biofilms, increased oxidative stress appears to result in part from down-regulation of enzymes that participate in the cellular defence to reactive oxygen species (ROS). To examine whether a reduction in the ability of sessile staphylococci to detoxify ROS could explain increased mutability of the staphylococcal biofilm, we performed transcriptome analysis by DNA microarray to look for de-regulation of genes implicated in ROS protection/detoxification in 48 and 144 hr biofilms of SH1000 compared to planktonic cultures (Table 1). Transcriptional profiles of 48 hr biofilm cultures showed numerous similarities with previously published expression data for *S. aureus* biofilms (Table 1) [25], including differential expression of SAOUHSC_01216 (*sucC*), SAOUHSC_01418 (*odhA*) and SAOUHSC_02350 (*atpB*). We noted up-regulation of at least two genes whose products are involved in protection against oxidative stress, namely *sodA* (SAOUHSC_01653), encoding superoxide dismutase, and *qoxA* (SAOUHSC_01002), encoding quinol oxidase subunit [26], [27]. Enhanced expression of *sodA,* following 48 hr biofilm growth, was confirmed using qRT-PCR (1.91-fold), although no differential expression of *qoxA* expression was detected (*data not shown*). Increased expression of antioxidant enzymes, such as SodA, in the biofilm further corroborates the idea that sessile staphylococci are subject to increased levels of oxidative stress, but suggests that increased oxidative stress is not the result of a reduced ability of staphylococci to detoxify ROS. We also performed transcriptional profiling of *S. aureus* biofilms grown for 144 hrs; to our knowledge, no transcriptional analyses are available in the published literature for *S. aureus* biofilms grown for longer than 48 hrs. However, the transcriptional profile of 144 hr biofilms exhibited little differential gene expression compared with planktonic cultures (Table 1).

**Table 2:** Bacterial strains used in this study.

Strain	Comments	MIC (µg/ml)		MIC (mg/ml)		Source
		RIF	MUP	VAN	ASC	
*S. aureus* SH1000	Standard laboratory strain	0.008	0.125	4	0.125	[46], [47]
*S. aureus* UAMS-1	Biofilm proficient strain	0.016	0.125	4	0.125	[23]
*S. epidermidis* RP62A	Biofilm proficient strain (ATCC35984)	0.016	0.125	nd	nd	ATCC
*S. aureus* KC043	KatA/AhpC defective derivative of SH10000	0.032	nd	nd	nd	[48]
*S. aureus* MHK11AM	SodA/SodM defective derivative of SH1000	0.032	nd	nd	nd	[35]
*S. aureus* USA300Δ*cidC*	CidC defective derivative of USA300 (strain NE564)	nd	nd	nd	nd	NARSA
*S. aureus* SH1000Δ*cidC*	CidC defective derivative of SH1000	0.008	nd	nd	nd	This study

RIF – Rifampicin, MUP – Mupirocin, VAN – Vanillin, ASC – Ascorbic acid. nd – Not determined. NARSA – Network on Antimicrobial Resistance in *Staphylococcus aureus*.

To further examine a potential role for antioxidant enzymes in the biofilm mutability of *S. aureus*, mutation frequencies for planktonic and biofilm cultures of the antioxidant enzyme-deficient strains *S. aureus* MHK11AM (SH1000 lacking the superoxide dismutases, SodA and SodM) and KC043 (SH1000 lacking the peroxidases, KatA and AhpC) were determined using rifampicin selection and compared with the wild-type. Although loss of these enzymes resulted in a further increase in biofilm mutability compared with *S. aureus* SH1000 (MHK11AM –2.2-fold, KC043–8.3-fold), a comparable increase in mutability was also observed in planktonic culture (MHK11AM –2.9-fold, KC043–10.8-fold). Since strains lacking antioxidant enzymes exhibited the same increased mutability in biofilms over planktonic culture as the wild-type strain, it appears that these enzymes are not involved in the phenomenon of biofilm mutability.

### Investigating a Role for Hydrogen Peroxide in Staphylococcal Biofilm Mutability

*S. pneumoniae* exhibits increased mutability during the biofilm mode of growth [12]. This phenomenon has been attributed to the activity of the pyruvate oxidase, SpxB [12], an enzyme that catalyzes the formation of acetyl phosphate from pyruvate, yielding the ROS hydrogen peroxide as a by-product. Since *S. aureus* encodes a pyruvate oxidase (CidC) with 33% identity to SpxB, we investigated the possibility that endogenous hydrogen peroxide may also be involved in staphylococcal biofilm mutability. Addition of catalase to biofilms of SH1000 reduced mutation frequencies (Figure 5), indicating that hydrogen peroxide indeed drives enhanced mutability in this strain during the biofilm mode of growth. In contrast, catalase did not significantly reduce the mutability of UAMS-1 biofilms *(data not shown)*. To assess the role of the CidC pyruvate oxidase in SH1000 biofilm mutability, the mutation frequencies of SH1000Δ*cidC* biofilms were determined. However, disruption of *cidC* in SH1000 caused no significant reduction in the mutability of biofilms (Figure 5).

## Discussion

Mutation plays an important role in the development of antibiotic resistance in *S. aureus* and other staphylococci [28]. Single point mutations in the chromosome can cause clinically-significant levels of resistance to a variety of antistaphylococcal agents, including rifampicin, mupirocin, fusidic acid and fluoroquinolones [28], whilst the accumulation of mutations at multiple loci can lead to resistance to glycopeptides and daptomycin [29], [30]. Any factor that elevates the basal mutation frequency will serve to accelerate the emergence of antibiotic resistance; we show here that growth as a biofilm appears to represent one such factor for the staphylococci.

Across a series of experiments using different staphylococcal species and strains, selecting agents and biofilm models, we consistently observed enhanced mutability in biofilm cultures versus planktonic cultures, with increases in mutation frequency ranging between ∼2-fold and ∼60-fold. At the upper end of this range, the mutation frequencies are comparable to those exhibited by hypermutator strains of *S. aureus* defective for DNA repair [31]. Although a number of variables appear to affect the level of mutability observed in staphylococcal biofilms, biofilm maturity seems particularly important; the greatest increases in mutability were associated with biofilms that had been established for ≥96 hrs (Figure 2).

Although the increases in mutation frequencies of staphylococcal biofilm populations were in most cases relatively modest, this phenomenon may nonetheless have considerable impact on the development of antibiotic resistance in *S. aureus* and other staphylococci. This is particularly the case since staphylococcal infections frequently include a biofilm component [13], [14], [15], [32]. Furthermore, since biofilm cells are protected from antibiotic-mediated killing, bacteria within the biofilm will benefit not only from an elevated mutation frequency to antibiotic resistance, but will also experience extended periods of drug selection. Staphylococcal biofilms may therefore represent an important site for the development of antibiotic resistant mutants, which could then become dispersed from the biofilm during the dissemination phase of biofilm growth [33].

Loss of the increased mutability upon incorporation of antioxidants into biofilms indicates that staphylococci in biofilms are more mutable because of oxidative stress. This idea is supported by the transcriptional analysis of 48 hr biofilms, which revealed up-regulation of genes encoding antioxidant enzymes, such as SodA, compared with planktonic cultures. Furthermore, it is likely that this oxidative stress is generated within the staphylococcal cell, as *sodA* expression is responsive to endogenous, rather than exogenous, superoxide [34], [35]. Consistent with this hypothesis it has also been suggested that *P. aeruginosa* is subject to endogenous oxidative stress during biofilm growth [24].

Enhanced mutability in *S. pneumoniae* biofilms has been attributed to production of hydrogen peroxide by the pyruvate oxidase, SpxB. Although biofilm mutability in *S. aureus* SH1000 was also attributed to the presence of hydrogen peroxide, this was not the result of the staphylococcal pyruvate oxidase, CidC, suggesting that another metabolic source of hydrogen peroxide probably accounts for the increase in mutation frequency in this strain. The inability of catalase to affect the mutability of *S. aureus* UAMS-1 biofilms suggests that there is more than one mechanism by which the mutability of staphylococci may increase during the biofilm mode of growth.

In conclusion, we have shown that staphylococcal biofilms exhibit an increase in basal mutation frequency as a consequence of increased oxidative stress, a phenomenon which will accelerate the emergence of antibiotic resistance in this genus.

## Materials and Methods

### Planktonic Growth of Bacterial Strains and Antibiotic Susceptibility Determinations

Strains used in this study are listed in Table 2. Antibiotic susceptibility testing was performed by agar dilution following the guidelines of the British Society for Antimicrobial Chemotherapy (BSAC), but used Mueller-Hinton broth and agar (MHB and MHA) in place of Iso-Sensitest medium [36]. All chemicals were from Sigma-Aldrich (Dorset, UK), with the exception of mupirocin and vanillin which were from USP Reference Standards (Rockville, USA) and MP Biomedicals (Illkirch, France), respectively. Planktonic cultures were grown for 18 hrs at 37°C with shaking in MHB or brain-heart infusion broth (BHB). Where required, antioxidants were added to cultures at ¼×MIC.

### Cellulose Disk Static (CDS) Biofilm Model

Mixed cellulose-ester membrane filter disks (25 mm diameter, 0.22 µm pore size; Millipore, Billerica, USA) were used as a substratum for the formation of staphylococcal biofilms. To promote biofilm formation, disks were immersed in 4% v/v normal pooled human plasma (Sera Laboratories International, Bolney, UK) diluted in 0.05 M carbonate buffer [37] and incubated at 4°C overnight prior to use. Plasma-soaked disks were immersed in a saturated culture of stationary phase bacteria, placed on brain heart infusion agar (BHA), and incubated at 37°C. To harvest cells from *S. aureus* biofilms for mutation frequency determinations, loosely-associated cells were first removed from the disks by gentle agitation in sterile saline, and the disks then incubated for 30 mins at 37°C in the presence of cellulase (1 mg/ml in 0.05 M citrate buffer pH 5.0) [38]. In the case of *S. epidermidis* biofilms, cells were liberated from the disks by incubation in phosphate-buffered saline pH 7.4 containing 625 µM sodium metaperiodate, 3.125 mM sodium acetate for 60 mins at 37°C [39]. In both cases, detached cells were then washed in sterile saline, harvested by centrifugation for 10 mins at 5000×*g*, and resuspended in sterile saline before proceeding to mutation frequency determinations as described below. Where required, biofilm cultures were supplemented with antioxidants (ascorbic acid, vanillin and catalase) by incorporation into BHA and by addition drop-wise directly to the cell population. Ascorbic acid and vanillin were added at ¼×MIC (0.03 mg/ml and 1 mg/ml, respectively); catalase was added at 4 U/ml. Methodology regarding the development and validation of this biofilm model can be found in Information S1.

### Sorbarod (Constant Flow) Biofilm Model

Inoculation, incubation and harvesting of biofilms on compacted cellulose (Sorbarod) filters were performed essentially as previously described [11]. To promote biofilm formation, Sorboarod filters were pre-treated with 1 ml of 4% (v/v) human plasma for 16 hrs at 4°C. Cells were harvested by vigorously vortexing the filters, followed by sonication for 15 mins.

### Mutation Frequency Determinations

Mutation frequencies were determined essentially as described by O’Neill *et al* [40]. Bacterial suspensions containing ∼10^9^ cfu/ml were spread onto selective MHA containing 4×MIC (Table 2) of rifampicin or mupirocin to recover spontaneous antibiotic-resistant mutants. Culture dilutions containing ∼10^2^ cfu/ml were spread onto non-selective MHA to determine viable cell numbers. Agar plates were incubated for 48–72 hrs at 37°C, and mutation frequencies expressed as the number of antibiotic-resistant mutants recovered as a proportion of the total cell count. Errors were calculated as 95% confidence intervals using Fieller’s Theorem [41]. Mutation frequencies were determined for six experimental replicates, each consisting of three biological replicates.

### Transcriptional Profiling by DNA Microarray

Planktonic cultures of SH1000 were combined with two volumes of RNAprotect Bacteria Reagent (Qiagen, Crawley, UK), and the cells harvested by centrifugation at 5000×*g*. CDS biofilm cultures of SH1000 were immersed in RNAprotect, detached with buffered cellulase and then centrifuged at 5000×*g* to pellet cells. Cells were washed in RNAprotect and stored at −80°C until required. Pellets were thawed at room temperature, washed in 10 ml of TE buffer (pH 7.4), resuspended in 1 ml of TE buffer (pH 7.4) containing 200 µg lysostaphin/ml (Sigma-Alrich, Dorset, UK), and incubated at 37°C for 60–90 mins. Proteinase K (40 µg/ml) (Sigma-Alrich, Dorset, UK) was then added to the mixture, and incubated continued for 10 mins at room temperature. Total RNA was purified from these samples using the RNeasy Midi kit (Qiagen) following the manufacturer’s instructions, including an on-column DNase digestion using the RNase-free DNase kit (Qiagen). cDNA synthesis, cDNA labelling, hybridisation using 4×72K multiplex microarrays, and quantification of gene expression were performed by Roche Nimblegen (Madison, USA). Of the 2892 predicted protein coding open reading frames of the *S. aureus* NCTC8325 genome, 2887 were represented on the microarrays. Data were analysed using ArrayStar™ (DNASTAR, Madison, USA), and genes were considered differentially regulated if the average gene expression value showed ≥2-fold increase or decrease with a *P* value of ≤0.05 by student’s *t* test [42], [43]. Transcriptional profiling was performed for three biological replicates, and the data deposited at the NCBI Gene Expression Omnibus under Accession GSE35837.

To confirm the transcriptional profiling data, real-time quantitative reverse transcription polymerase chain reaction (qRT-PCR) was performed. Synthesis of cDNA using total RNA (RT) and qPCR were performed using the Quantitect RT kit and Quantitect SYBR Green PCR kit (Qiagen), respectively, according to the manufacturer’s instructions. The relative quantities of *gyrA* (endogenous control) and *sodA* mRNA transcripts in planktonic and 48 hr biofilm cultures were determined by qPCR using primers GyrA_Fwd (5′-ATAGAAATGGTAAGATTGCGATT-3′), GyrA_Rev (5′-ACCTTTCACACCCGTTGC-3′), SodA_Fwd (5′-GCGCCAATGTAGTCAGGGCGTTTG-3′) and SodA_Rev (5′-TGCACGCTTTGGTTCAGGTTGGG-3′) by relative standard curve quantification. Data were analysed using MxPro Mx3005P software (Agilent, Wokingham, UK). Relative expression levels of *sodA* were calculated with respect to *gyrA* as the endogenous control. Data were regarded as significant if a *P* value = ≤0.05 was returned following a Student’s t-test.

### Inactivation of the cidC Gene in *S. aureus* SH1000

The transposon-disrupted *cidC* gene from *S. aureus* USA300Δ*cidC* (NE564; Nebraska transposon mutant library [44]) was transduced into *S. aureus* SH1000 using Φ11 [45], to generate *S. aureus* SH1000Δ*cidC*. Successful transduction of this locus to SH1000 was confirmed by PCR.

## Supporting Information

Information S1 **Methodology and additional information regarding optimisation and validation of the cellulose disk model.**
(DOCX)
