# Supplementary material for: Increased Mutability of Staphylococci in Biofilms as a Consequence of Oxidative Stress
Source: PLoS One. 2012 Oct 24;7(10):e47695. doi: 10.1371/journal.pone.0047695 (PMC3480535; doi:10.1371/journal.pone.0047695)
Supplement: Information S1 — Methodology and additional information regarding optimisation and validation of the cellulose disk model. (DOCX) [file pone.0047695.s001.docx]

**Supporting Information S1**

**Development of the cellulose disk biofilm model for staphylococci**

The methods and results below outline the optimisation and validation of the cellulose disk biofilm model.

**Methods (for Figure 1)**

**Susceptibility of planktonic and cellulose disk cultures to rifampicin (Figure 1C)**

Planktonic and cellulose disk cultures were challenged with 0.25 µg rifampicin/ml (representing 32x planktonic MIC). Planktonic cultures were cultured overnight and then diluted 1:100 in fresh brain heart infusion broth (BHB). These cultures were incubated at 37°C with shaking until an OD_600nm_ of 0.3 was reached, and the cells were then harvested by centrifugation and resuspended in 1/10^th^ volume BHB containing 0.25 µg rifampicin/ml. Cellulose disk cultures were grown for 48 hrs and then placed on BHA containing 0.25 µg rifampicin/ml. For both cultures types, drug-free control samples were also used. At intervals of 1 hr over a 5 hr period, planktonic culture samples and resuspended disk cultures (as described in the cellulose disk model methodology) were serially diluted, plated onto MHA, incubated at 37°C for 24 hrs and the number of colonies enumerated.

**Effects of l- and d-tyrosine on cellulose disk cultures (Figure 1D)**

Cellulose disk cultures grown for 48 hrs were washed in saline. Washed biofilms were then incubated with 100 µM l- or d-tyrosine (dissolved in PBS), for 30 mins at 37°C with shaking. Disk cultures were then subjected to vortex mixing for 1 min and the supernatants collected. The disks were then removed from the amino acid solutions and incubated with cellulase (as described in the cellulose disk model methodology). Amino acid- and cellulase‑liberated cells were then serially diluted and plated onto MHA and colonies enumerated after 24 hrs of incubation at 37°C.

**Results (for Figure 1)**

**Optimisation of the cellulose disk biofilm model**

Mixed cellulose ester membrane filter disks (25 mm diameter, 0.22 μm pore size, Millipore) were used to evaluate whether this substratum promotes the formation of staphylococcal biofilms. Initially, the cellulose disk model was optimised for use with *S. aureus* SH1000 [[1](#_ENREF_1)]. Human serum components, such as fibronectin and fibrinogen, promote the adherence of staphylococci to surfaces [[2](#_ENREF_2),[3](#_ENREF_3)]. To exploit this, human plasma (Normal pooled human plasma, Sera Laboratories International), diluted in 0.05 M carbonate-bicarbonate buffer, was added to the disks prior to inoculation. Inoculation was achieved by briefly immersing the disks in stationary phase cultures. The minimum concentration of human plasma that promoted maximum adherent viable cell numbers was determined using disk cultures incubated for 48 hrs at 37°C (Figure 1A). Loosely-associated nonadherent bacteria were removed from disk cultures by washing in saline with gentle agitation (10 mins). Adherent cells were enumerated following detachment by treatment with cellulase (1 mg/ml in 0.05 M citrate buffer) [[4](#_ENREF_4),[5](#_ENREF_5)]. The minimum effective concentration of human plasma was 4% (v/v), resulting in 2 x 10^10^ adherent cfu/disk (Figure 1A). The addition of human plasma to the system increased the proportion of adherent cells from ~10% to ~93% (Figure 1B).

To achieve prolonged culture using this model it was necessary to submerge disk cultures in 4% (v/v) human plasma and place them onto fresh BHA every 48 hrs. This process significantly improved the yield of adherent cells from ~16% to ~84% in disk cultures grown for 144 hrs (Figure 1B). This model was also effective for generating adherent cultures of *S. aureus* UAMS-1 [[6](#_ENREF_6)] and *Staphylococcus epidermidis* RP62A (ATCC35984) (data not shown). *Staphylococcus epidermidis* RP62A, required the use of sodium metaperiodate for detachment.

**Evidence that bacteria in the cellulose disk model grow as biofilms**

Several experiments were conducted to confirm that this system generated biofilms of staphylococci. Recalcitrance to antibiotics is one of the most extensively documented characteristics of biofilms [[7](#_ENREF_7)]. Accordingly, time kill curves were determined for planktonic cultures and adherent bacteria grown using this system (Figure 1C). Specifically, exponential phase planktonic cultures were concentrated 10-fold and exposed to rifampicin (0.25 μg/ml). In a similar manner, adherent cultures were grown for 48 hrs, placed on BHA containing 0.25 µg/ml rifampicin, and then harvested as described above. Rifampicin reduced the number of viable cells in planktonic cultures by 2 log_10_ units after 5 hrs of incubation. Adherent cultures demonstrated no significant reduction in viable cell numbers over the same time period.

d-amino acids disassemble or inhibit the formation of biofilms formed by several bacterial species, including staphylococci, a phenomenon not observed in the presence of the l‑enantiomers [[8](#_ENREF_8)]. The dissociation of adherent *S. aureus* SH1000 cultures in the presence of d- and l-tyrosine (100 µM in PBS for 30 mins at 37°C) was therefore assessed (Figure 1D), followed by incubation in cellulase for detachment of the remaining adherent cells (as described above). This revealed ~71% and ~7% dissociation of the adherent population by the d- and l‑enantiomers, respectively.

Following model optimisation and the validation described above, atomic force microscopy (AFM) and confocal microscopy were used to visualise bacteria grown in this system. AFM and confocal images of *S. aureus* SH1000, grown for 48 hrs, revealed closely packed cells with a rough and undulating surface structure (*data not shown*).

Taken together, these investigations, as well as the microarray data discussed in the main paper, demonstrate that the cellulose disk model supports the growth of staphylococci as biofilms.

**References**

1. Horsburgh MJ, Aish JL, White IJ, Shaw L, Lithgow JK, et al. (2002) σ^B^ modulates virulence determinant expression and stress resistance: Characterization of a functional *rsbU* strain derived from *Staphylococcus aureus* 8325-4. J Bacteriol 184: 5457-5467.

2. Keane FM, Loughman A, Valtulina V, Brennan M, Speziale P, et al. (2007) Fibrinogen and elastin bind to the same region within the A domain of fibronectin binding protein A, an MSCRAMM of *Staphylococcus aureus*. Mol Microbiol 63: 711-723.

3. O'Brien L, Kerrigan SW, Kaw G, Hogan M, Penades J, et al. (2002) Multiple mechanisms for the activation of human platelet aggregation by *Staphylococcus aureus*: roles for the clumping factors ClfA and ClfB, the serine-aspartate repeat protein SdrE and protein A. Mol Microbiol 44: 1033-1044.

4. Cescutti P, Toffanin R, Fett WF, Osman SF, Pollesello P, et al. (1998) Structural investigation of the exopolysaccharide produced by *Pseudomonas flavescens* strain B62 - degradation by a fungal cellulase and isolation of the oligosaccharide repeating unit. Eur J Biochem 251: 971-979.

5. Loiselle M, Anderson KW (2003) The use of cellulase in inhibiting biofilm formation from organisms commonly found on medical implants. Biofouling 19: 77-85.

6. Gillaspy AF, Hickmon SG, Skinner RA, Thomas JR, Nelson CL, et al. (1995) Role of the accessory gene regulator (*agr*) in pathogenesis of staphylococcal osteomyelitis. Infect Immun 63: 3373-3380.

7. Ito A, Taniuchi A, May T, Kawata K, Okabe S (2009) Increased antibiotic resistance of *Escherichia coli* in mature biofilms. Appl Environ Microbiol 75: 4093-4100.

8. Kolodkin-Gal I, Romero D, Cao S, Clardy J, Kolter R, et al. (2010) D-amino acids trigger biofilm disassembly. Science 328: 627-629.
